# Analyzing Factors Affecting Emergency Department Length of Stay—Using a Competing Risk-accelerated Failure Time Model

**DOI:** 10.1097/MD.0000000000003263

**Published:** 2016-04-08

**Authors:** Chung-Hsien Chaou, Te-Fa Chiu, Amy Ming-Fang Yen, Chip-Jin Ng, Hsiu-Hsi Chen

**Affiliations:** From the Department of Emergency Medicine (C-HC, T-FC, C-JN), Chang Gung Memorial Hospital, Linkou and Chang Gung University College of Medicine, Taoyuan, Taiwan; Institute of Epidemiology and Preventive Medicine (C-HC, H-HC), College of Public Health, National Taiwan University, Taipei, Taiwan; and School of Oral Hygiene (AM-FY), College of Oral Medicine, Taipei Medical University, Taipei, Taiwan.

## Abstract

Emergency department (ED) length of stay (LOS) is associated with ED crowding and related complications. Previous studies either analyzed single patient disposition groups or combined different endpoints as a whole. The aim of this study is to evaluate different effects of relevant factors affecting ED LOS among different patient disposition groups.

This is a retrospective electronic data analysis. The ED LOS and relevant covariates of all patients between January 2013 and December 2013 were collected. A competing risk accelerated failure time model was used to compute endpoint type-specific time ratios (TRs) for ED LOS.

A total of 149,472 patients was included for analysis with an overall medium ED LOS of 2.15 [interquartile range (IQR) = 6.51] hours. The medium LOS for discharged, admission, and mortality patients was 1.46 (IQR = 2.07), 11.3 (IQR = 33.2), and 7.53 (IQR = 28.0) hours, respectively. In multivariate analysis, age (TR = 1.012, *P* < 0.0001], higher acuity (triage level I vs level V, TR = 2.371, *P* < 0.0001), pediatric nontrauma (compared with adult nontrauma, TR = 3.084, *P* < 0.0001), transferred patients (TR = 2.712, *P* < 0.0001), and day shift arrival (compared with night shift, TR = 1.451, *P* < 0.0001) were associated with prolonged ED LOS in the discharged patient group. However, opposite results were noted for higher acuity (triage level I vs level V, TR = 0.532, *P* < 0.0001), pediatric nontrauma (TR = 0.375, *P* < 0.0001), transferred patients (TR = 0.852, *P* < 0.0001), and day shift arrival (TR = 0.88, *P* < 0.0001) in the admission patient group.

Common influential factors such as age, patient entity, triage acuity level, or arrival time may have varying effects on different disposition groups of patients. These findings and the suggested model could be used for EDs to develop individually tailored approaches to minimize ED LOS and further improve ED crowding status.

## INTRODUCTION

Emergency medicine, now recognized as an essential part of public health service, has developed rapidly since its founding 40 years ago. As the services provided by emergency departments (EDs) increase and the management process becomes more complicated, patients stay in EDs for longer and EDs become more crowded.^1^ A number of studies have discussed the adverse impacts of ED crowding, which include prolonged waiting times, increased complications, and increased mortality.^2–5^ Previous literature has also demonstrated that prolonged ED length of stay (LOS) is not only a cause but also a result of ED crowding, yielding a vicious cycle.^6,7^ Therefore, it is worthwhile to elucidate the factors associated with ED LOS in order to alleviate ED crowding and improve quality of care.

Many factors are responsible for ED LOS. Recent studies have shown that increased testing, consultation, radiology studies, and provision of less substantial treatment cause a significant increase in ED LOS.^8–10^ Disease and acuity factors, including higher triage level, certain presenting symptoms, or delayed pain alleviation have also been associated with prolonged ED LOS.^11^ Some previous studies suggested that demographic characteristics, such as age and ethnicity, or the presence of junior residents or medical students, are associated with longer ED LOS.^12–15^ Regarding the patient populations, some of the studies focused on patients who were admitted or discharged, whereas others analyzed ED patient populations by grouping different patient dispositions together. However, it is possible that some of the influential factors, such as consultation or triage acuity level, will have different effects on patients with different final dispositions.

In general, there are 3 final fates for ED patients: discharge, admission to the hospital, or expiring in the ED. It is difficult to differentiate the effects of certain factors on different endpoints if the analysis combines different dispositions as a unified group. It is also inadequate to analyze them separately, as a patient who expires in the ED while waiting for admission may be excluded from an analysis of the admitted patients group. The neglect of those deceased patients before admission would render the ED LOS underestimated. An alternative solution is to utilize the competing risk model for event-time analysis.^16,17^ A competing risk model allows researchers to classify events into different types and to estimate type-specific hazards simultaneously. The aim of this study is to investigate factors that influence ED LOS according to different endpoint types.

## METHODS

### Study Design

A retrospective analysis of the administrative database was conducted in a 12-month study period. The study protocol was determined before the data were collected. The study protocol was also approved by the local ethics review board and was exempt from the requirement of obtaining informed consent.

### Study Setting and Population

This study was conducted in the ED of Linko Chang-Gung Memorial Hospital, a teaching hospital, tertiary medical center, and level I trauma center with a 3600-bed capacity and annual ED visits of approximately 150,000 patients. All patients who had registered in the ED from January 2013 to December 2013 were included for analysis. Patients with missing registration time or leaving time were excluded. The patient population consisted of local people with general emergency conditions as well as transfer cases from regional hospitals. Patients were divided into trauma, adult nontrauma, and pediatric nontrauma groups, and were managed in different areas within the ED. The demarcation between adult and pediatric nontrauma patients was the age of 18 years.

### Data Collection

Data were extracted from the hospital administrative electronic database. The endpoints were classified as discharged, admission, and mortality. The discharged patients included those who were discharged by the primary ED physician, those who left without being seen, and those who left unnoticed. The admission patients included those admitted to the intensive care unit (ICU), those admitted to a ward, and those who were transferred to another hospital for admission. Patient LOS was defined as the time from registration to departure, which was recorded at the registration counter. Patient characteristic variables included age and gender. The disease and acuity variables included patient entity, triage level, whether the patient was transferred from another hospital, whether the patient was in an out of hospital cardiac arrest condition, and whether the primary ED physician declared the patient to be in critical condition. The arrival time, stratified by 8-hour shifts, was also recorded. The triage classification was sorted using the criteria of the Taiwan Triage Acuity System, which is a 5-level system. All triage nurses were senior nurses who had attended a special triage training program.

### Statistical Analysis and Competing Risk Model

In the descriptive analysis, normality tests were performed for continuous variables. Mean and standard deviation (SD) were used to describe the central tendency and spread for continuous variables. Median and interquartile range (IQR) were used for continuous variables that obviously deviated from normal distribution. Comparisons of the variables between the 3 groups were made using analysis of variance (ANOVA), the Wilcoxon rank-sum test, or the Chi-square test, when appropriate.

For the event-time analysis, an accelerated failure time model was used to evaluate the dependence of ED LOS on relevant covariates. This model was chosen because of its intuitive direct modeling of the logarithm of survival times.^18^ Factors that affect ED LOS have a positive value for the time ratio (TR), which is equal to the reciprocal of the exponential of the estimated regression parameter. A TR smaller than 1 indicates that the factor reduces LOS, and a TR greater than 1 means that the factor lengthens LOS. The Weibull distribution of the survival time was determined after comparing the goodness-of-fit using the likelihood-ratio statistic. To cope with the existence of more than 1 type of endpoint and the fact that the occurrence of 1 type of event removes the individual from the risk set of all the other event types, a competing risk-accelerated failure time model was used.^19^ Type-specific survival curves and TRs were estimated while treating all other event types as censoring. For ordinal predictors, a log-rank trend test was used to discover any trends in magnitude of influence. All analyses were performed using SAS statistical software version 9.3 (SAS Institute Inc., Cary, NC).^20^ A reported *P* value of less than 0.05 was considered statistically significant.

## RESULTS

### Descriptive Analysis

**FIGURE 1:**
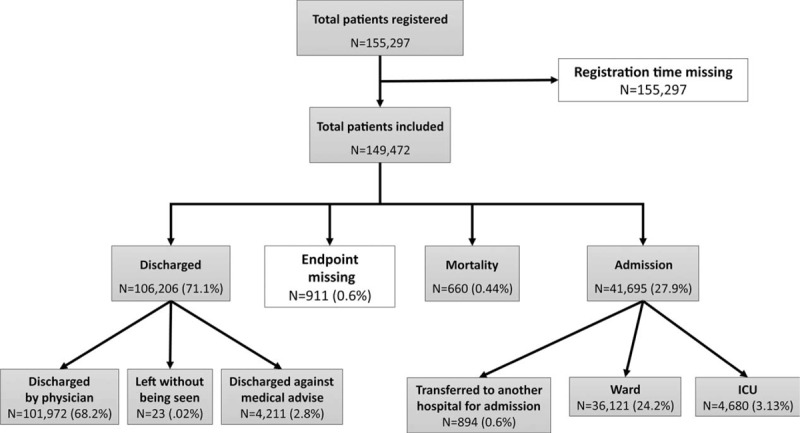
Flow chart of inclusion and exclusion process.

Data for 149,472 patients were analyzed after exclusion. There were 106,206 patients discharged from the ED, 41,695 patients admitted to the hospital, and 660 patients who died in the ED. Detailed patient inclusion and endpoints are shown in Figure 1. Table 1 displays the characteristics of all patients and each endpoint. The overall average patient age was 39.8 (SD = 27.1). The overall median LOS was 2.15 hours, with an IQR of 6.51 hours. The age group, patient entity, and triage level with the largest proportion were 40 to 60 years, adult nontrauma, and level 3, respectively.

**TABLE 1:**
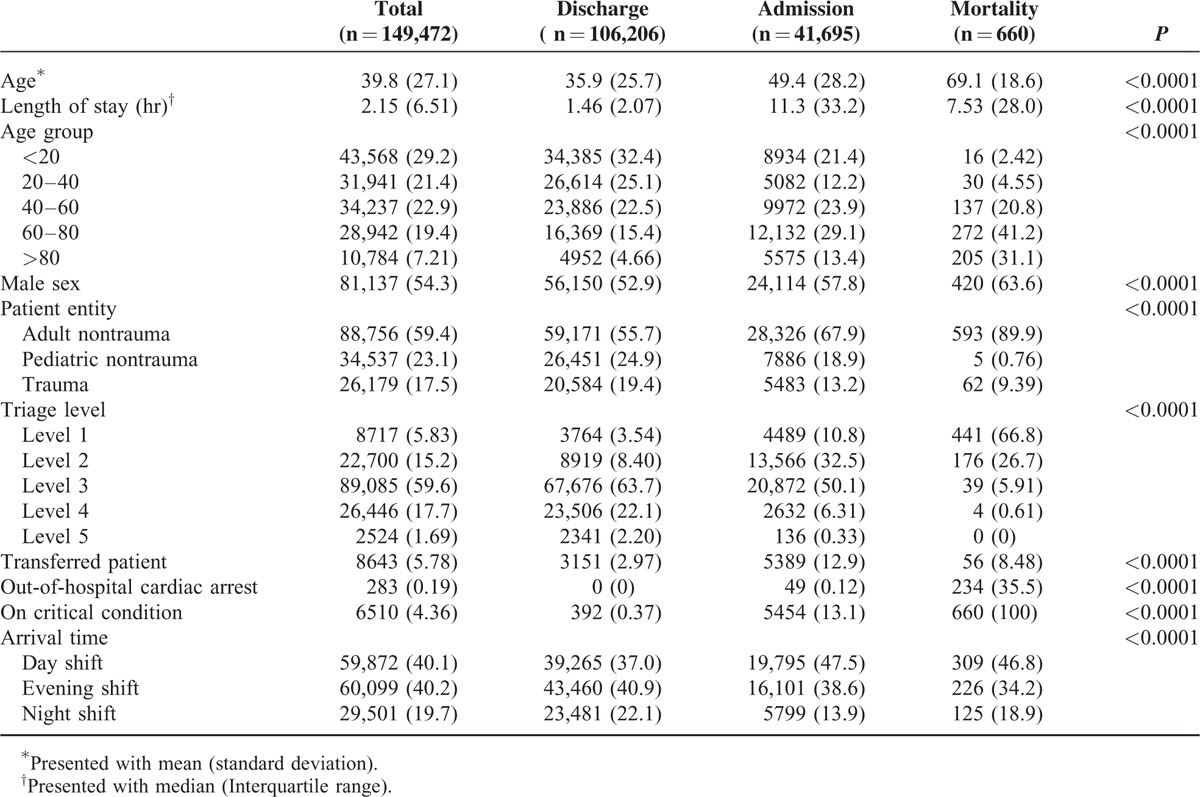
Baseline Characteristics of the Included Patients, Presented With Count (Percentage) Unless Stated Otherwise

### Differences Between Patient Endpoint Groups

**FIGURE 2:**
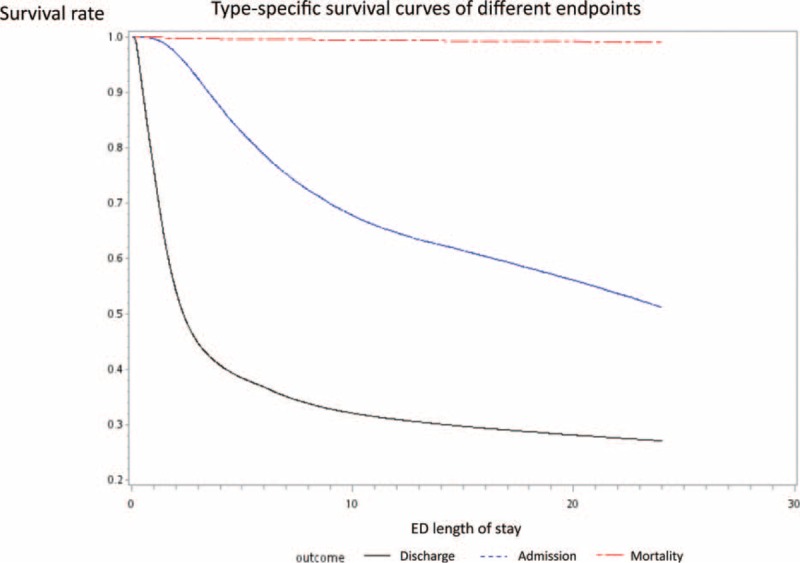
Type specific survival curves of different endpoints.

The disparities of the characteristics between patients with different endpoints are summarized in Table 1. The discharged patients tended to be younger (mean age = 35.9, SD = 25.7), had shorter LOS (median = 1.46 hours, IQR = 2.07 hours), and fewer transfers (2.97%). They presented with lower acuity (triage level III-V, 88.1%) and no out-of-hospital cardiac arrest patients. On the contrary, the expired patients were much older (mean age = 69.1, SD = 18.6), had longer LOS (median = 7.53 hours, IQR = 28.0 hours), and more transfers (8.48%). They also presented with higher acuity (triage level III-V, 6.52%) and more out-of-hospital cardiac arrest patients (35.5%). The characteristics of the admission patient group were in the middle of the other 2 groups, with the exception of possessing the longest LOS (median = 11.3 hours, IQR = 33.2), indicating a crowded queue waiting for admission and having the most transfer patients (12.9%). All the differences in basic characteristics between the groups were statistically significant. A plot type specific survival curve of the 3 different endpoints is shown in Figure 2.

### Results of the Competing Risk Model

The results of multivariate analysis on the effects of possible covariates on ED LOS are displayed in Table 2. As can be seen, in the discharged group, longer ED LOS was noted for the following: higher age (TR = 1.012, *P* < 0.0001); male sex (TR = 1.025, *P* = 0.0004); pediatric nontrauma patient entity (compared with adult nontrauma, TR = 3.084, *P* < 0.0001); higher acuity (triage level I vs level V, TR = 2.371, level II vs level V, TR = 2.461, both *P* < 0.0001); transferred patients (TR = 2.712, *P* < 0.0001); those with a critical condition declared by an ED physician (TR = 5.576, *P* < 0.0001); and day shift arrival (compared with night shift, TR = 1.451, *P* < 0.0001). In the admission group, adult nontrauma patients (compared with pediatric nontrauma patients, TR = 2.666, *P* < 0.0001) and night shift arrivals (compared with day shift, TR = 1.136, *P* < 0.0001) experienced longer ED LOS. Male sex (TR = 0.958, *P* < 0.0001), higher acuity (triage level I vs level V, TR = 0.532, level II vs level V, TR = 0.614, both *P* < 0.0001), transferred patients (TR = 0.852, *P* < 0.0001), and those with a critical condition declared (TR = 0.792, *P* < 0.0001) experienced shorter ED LOS. The results for the mortality group were similar to those for the admission group, except that adult nontrauma (compared with pediatric non-trauma, TR = 0.117, *P* < 0.0001) and night shift (compared with day shift, TR = 0.689, *P* = 0.0009) were associated with shorter ED LOS. Trend tests for the effect of different triage levels on the survival time of different endpoints were all significant (*P* < 0.0001).

**TABLE 2:**
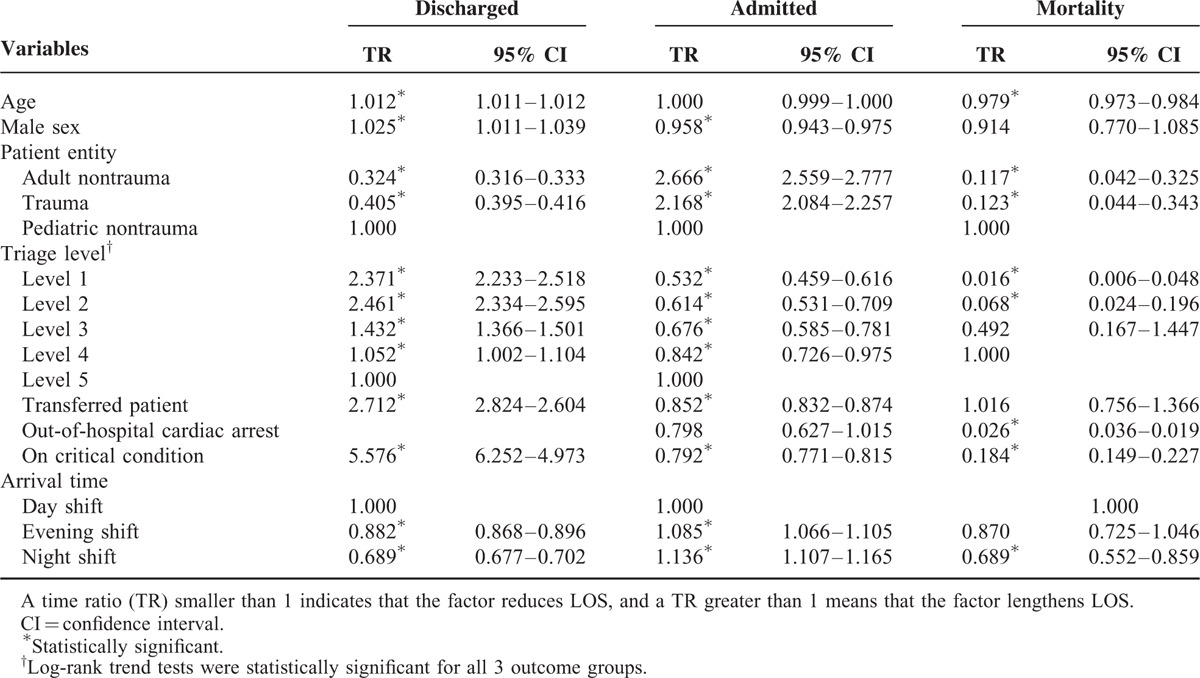
Multivariate Analysis on the Effects of Possible Covariates on Emergency Department Length of Stay (LOS), using a Competing Risk-accelerated Failure Time Model

## DISCUSSION

The current study demonstrated that the factors influencing ED LOS might have differential effects on different patient endpoint groups. To the authors’ knowledge, this is the first study to address this issue. Although the effects of these factors may not be universally applicable, the approach could be used in all EDs for local LOS analysis, and improvements could be planned according to individual results.

The majority of patients were discharged from the ED in the current study. Compared with previous studies, the ED discharge rate of 71% is at the lower end of previously reported values, which ranged from 70% to 90%.^1,21,22^ This may be due to the nature of a teaching hospital and medical center that treats more transferred or critically ill patients. The factors prolonging ED LOS for discharged patients included age, male sex, adult nontrauma patients (compared with pediatric nontrauma patients or trauma patients), higher triage acuity, transferred patients, those in critical condition, and day shift arrival (compared with evening or night shifts). Some of these factors are comparable with previous studies. Among them, triage level was most often mentioned in association with LOS, even in EDs with different triage systems.^11,23,24^ Age is another factor that was found to be related to increased ED LOS across different nations.^9,11,23,25^ Often, the elderly present with less specific symptoms and poor communication skills as well as more underlying conditions and comorbidities. These findings can give a clue to implementing interventions to improve patient ED LOS. For example, some reports have revealed improvements in ED LOS with the addition of an “ED fast track” for nonadmitted, lower acuity patients, without compromising waiting times and LOS for other ED patients.^26,27^ In another example, reported by Asha and Ajami,^28^ an early senior medical assessment and streaming model improves ED LOS in stable, ambulant senior patients.

**FIGURE 3:**
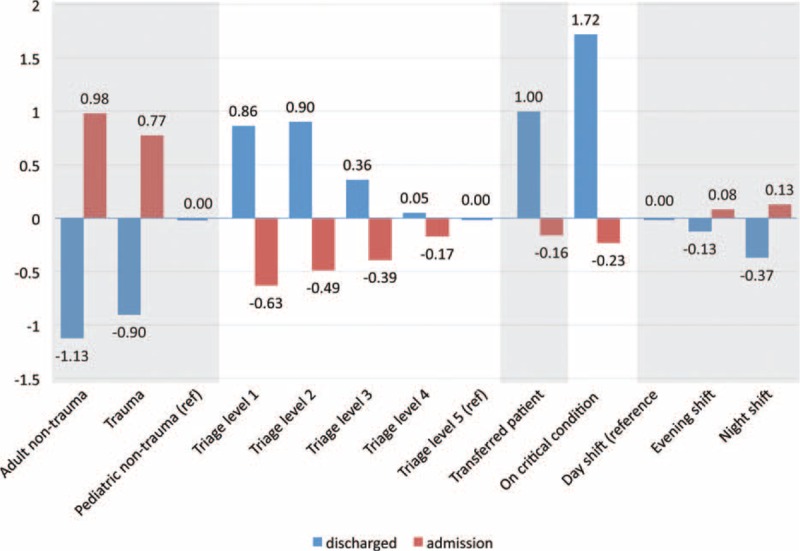
Comparison of regression coefficients between discharged and admission patient groups (comprised of 99% of all patients). A negative regression coefficient indicates that the factor reduces length of stay (LOS), and a positive regression coefficient represents that the factor lengthens LOS.

For the admitted patients, the median LOS was much longer than for the discharged patients. Some patients with special medical and socioeconomic conditions even stayed in the ED for more than 10 days. A large queue of patients waiting for admission was a significant factor in ED crowding and posed a heavy burden on the ED staff. As can be seen from the results, adult nontrauma patients who were waiting for medical wards tended to stay in the ED longer, reflecting the reality that chronic, geriatric medical diseases have gradually dominated the medical resources in an aging population like Taiwan. From the current study, it is very interesting to note that the influence of triage acuity on ED LOS varied with the endpoint used, but all 3 groups consistently showed a gradient relationship between triage level and ED LOS. Besides the triage acuity levels, the TRs for transferral and critical condition also yielded opposite results in the admission group compared with the discharged patient group (Figure 3). In other words, patients with higher acuity or in more critical condition had shorter ED LOS if they were admitted. This might be explained by the fact that the priority for admission within a specific medical specialty is ranked according to both the severity of the disease and the arrival time, and that ICU waiting times are generally shorter than ward waiting times. It is clear from the previous literature that the LOS of the admitted patients is determined not only by ED or patient factors but also by hospital-level determinants such as hospital occupancy, admission-discharge ratio, and the daily hospital and ICU census.^29–32^ Thus, some of the strategies developed to improve ED LOS for admission patients, such as creating specialized acute medical admission wards, increasing ICU capacity, arranging admission immediately after evaluation, or developing disease-specific protocols, may be very different from those for discharged patients.^33–37^

Analyzing the effect of arrival time on ED LOS showed that LOS for discharged patients and expired patients tended to be shorter on night shifts than on day shifts. In contrast, LOS tended to be longer for patients in the admission group who arrived during night shifts. This could be explained by the fact that the ED is often crowded during the day, and patients with lower acuity levels may wait longer to be treated and discharged. In addition, this hospital does not admit patients to wards during the night shift, so night shift arrivals need to wait until daytime to be admitted, thus prolonging the LOS. For expired patients, the recognition of serious conditions is often delayed at night, resulting in more critical situations on arrival at the ED. Some different results have been shown in previous studies. Bekmezian et al^13^ found that prolonged ED-LOS for admitted pediatric patients is associated with morning arrival. Another study by Nelson et al^24^ reported that night shift arrival is associated with a LOS of more than 10 hours. The discrepancy may be caused by distinct arrival patterns and staffing policies within each hospital or region. This information can be valuable at the administrative level for planning provider allocations according to different patient populations and arrival times. Good examples of staffing management guided by statistical modeling are also found in the literature.^38^

## LIMITATIONS

There are several limitations to the current study. First, this was a single-center study and the results may not apply to another hospital with a different patient population. However, it was not the authors’ intention to develop a universally employable result regarding the influential patterns of all possible factors, but rather to reveal the different effects of these factors on different patient disposition groups. Second, not all of the possible correlates that affect ED LOS presented in previous literature were incorporated; some of the factors mentioned above, such as ethnicity, hospital occupancy, and presentation of medical students, were not included in the analysis because our dataset did not contain these information.

## CONCLUSION

In the present study, the authors analyzed the factors affecting ED LOS between different patient disposition groups using a competing risk-accelerated failure time model. The results showed that common influential factors such as age, patient entity, triage acuity level, or arrival time might have different effects on different disposition groups of patients. These findings and the suggested model could be used for EDs to develop individually tailored approaches to minimize ED LOS and further improve ED crowding status.
